# Effect of Solute Concentration and Filtration Rate on the Scale Production of a Physically Stable Amorphous Solid Form of Nilotinib

**DOI:** 10.3390/pharmaceutics17080998

**Published:** 2025-07-31

**Authors:** Zhihui Yuan, Bowen Zhang, Asad Nawaz, Zunhua Li

**Affiliations:** 1 Hunan Engineering Technology Research Center for Comprehensive Development and Utilization of Biomass Resources, College of Chemistry and Bioengineering, Hunan University of Science and Engineering, Yongzhou 425199, China; zhh_yuan@huse.edu.cn (Z.Y.); 007298@yzu.edu.cn (A.N.); 2 School of Chemical Engineering and Technology, Tianjin University, Tianjin 300072, China; bowen_667@tju.edu.cn

**Keywords:** physical stability, neutralization precipitation, solute concentration, filtration rate

## Abstract

**Background/Objectives:** Amorphous solid drugs exhibit physical instability and a propensity for crystallization, which leads to reduced solubility and bioavailability. Hence, this study optimized scale manufacturing parameters for producing a physically stable amorphous solid form of nilotinib using neutralization precipitation. **Methods:** A systematic evaluation of the effects of the solute concentration and filtration rate on amorphous physical stability was conducted using the pair distribution function (PDF), principal component analysis (PCA), and reduced crystallization temperature (R_c_) values. **Results:** It showed concentration-dependent crystallization resistance, with optimal physical stability achieved at a solute concentration of 0.126 mol/L and a 124 mL/min filtration rate. Experiments carried out at a scale of 50 g confirmed the stability of the production process. **Conclusions:** These findings provide a validated framework for developing lab-scale amorphous drug products with improved shelf-life stability, assessed using indirect indicators (PDF, R_c_) and confirmed through accelerated stability tests.

## 1. Introduction

In pharmaceutical formulation design, optimizing amorphous systems emerges as a critical strategy, notably for overcoming bioavailability limitations in solubility-challenged drugs. Amorphous formulations are characterized by their non-crystalline molecular architecture, which imparts enhanced aqueous solubility and rapid dissolution kinetics compared to their crystalline counterparts. This structural distinction enables improved biopharmaceutical performance through accelerated drug release profiles. This characteristic is especially vital for addressing the issue of low aqueous solubility that affects approximately 40% of newly developed drugs [1,2]. Therefore, amorphous solid dispersions are commonly employed to exploit this metastable state, where drug molecules are embedded into polymer matrices to preserve the physical stability of the amorphous solids [3,4]. Nevertheless, the thermodynamic instability inherent in these systems poses formulation challenges, as spontaneous crystallization triggered by factors such as heat, moisture, or mechanical stress can adversely affect the clinical performance of the drugs [5,6,7]. Current research endeavors are directed towards the development of polymer-stabilized matrices and the incorporation of advanced methodologies, including atomic resolution microscopy and solid-state nuclear magnetic resonance spectroscopy, to elucidate the pathways of crystallization and the molecular mobility of drugs [8,9]. The application of these technologies enables the rational design of enhanced amorphous drug systems with improved physical stability characteristics.

Furthermore, understanding the physical properties related to amorphous forms is critically important. Nonetheless, the preservation of this amorphous state throughout the processes of manufacturing, storage, and administration poses challenges [10]. Consequently, a significant volume of ongoing research is dedicated to the formulation of innovative strategies aimed at stabilizing the amorphous state of drugs, thereby ensuring their long-term efficacy. Various primary methodologies for the preparation of amorphous drugs each present distinct advantages and challenges, including anti-solvent precipitation, solvent evaporation, spray drying, hot melt extrusion, ball milling, and neutralization precipitation. For example, recent investigations have indicated that through the meticulous optimization of parameters, neutralization precipitation can yield physically stable amorphous formulations. This is exemplified by research on nilotinib, where the amorphous form produced under optimal conditions exhibited markedly enhanced physical stability [11]. In addition, multiple factors in anti-solvent precipitation, such as residual solvent content, drying duration, solvent/anti-solvent ratio, injection rate, mixing velocity, aging period, temperature, and cake thickness, jointly determine the physical stability of the formed amorphous materials [12,13,14]. Therefore, a thorough understanding and regulation of these parameters are critical for sustaining the physical stability and efficacy of amorphous drugs.

Additionally, preliminary research findings suggest that the utilization of the pair distribution function (PDF) derived from the Fourier transformation of powder X-ray diffraction (PXRD) data can effectively characterize the degree of molecular disorder within amorphous samples, with samples demonstrating greater disorder correlating with improved physical stability [12,13,14,15,16,17]. For example, Bøtker et al. employed PDF analysis to optimize the cryo-milling time for maximizing disorder and stability in γ-indomethacin samples [16]. Similarly, previous research has applied PDF to associate the diminished stability of amorphous piroxicam with the residual structural order [17]. These studies demonstrate that comparative analysis of G(r) values between amorphous and crystalline phases via PDF effectively evaluates disorder levels. The improved concordance of G(r) between amorphous and crystalline states suggests a greater structural similarity, reflecting the preservation of organization within the amorphous material. Additionally, the decreased crystallization temperature (R_c_) serves as a complementary measure for assessing the kinetic stability of amorphous substances. This thermal parameter evaluates the material’s vulnerability to transitioning into a crystalline phase, with higher R_c_ values being directly associated with increased energy barriers that inhibit unwanted crystallization [11,12,13,14].

This study examines nilotinib, an anticancer drug, as a representative compound. The crystalline structure of nilotinib demonstrates restricted solubility, which subsequently impedes its bioavailability. In response to this challenge, various formulation strategies have been investigated to improve solubility, including the creation of amorphous solid dispersions and nanoparticles through spray drying methods [18,19,20]. The principal aim of this investigation is to prepare an amorphous form of nilotinib using a neutralization precipitation technique, with a focus on optimizing the preparation process by adjusting the concentration of nilotinib and the filtration rate. This approach seeks to produce amorphous solids that exhibit improved physical stability. The physical stability of the obtained amorphous materials was assessed by analyzing PDF and R_c_ [11,12,13,14,15,21]. Additionally, this research employed principal component analysis (PCA) as a statistical methodology to facilitate the clarification of the discrepancies observed in the PDF data of different solid samples. In addition, studies investigated the scalability effects of nilotinib production by assessing how varying manufacturing scales influenced the amorphous solid-state stability of the active material. Finally, the accelerated stability test was performed to verify the long-term stability of the amorphous solid forms. This study provides a comprehensive examination of various facets related to amorphous drugs, encompassing their preparation, characterization, stability evaluation, and scale production, thereby offering significant insights for the future advancement of amorphous drug formulations.

## 2. Materials and Methods

### 2.1. Materials

**Figure 1:**
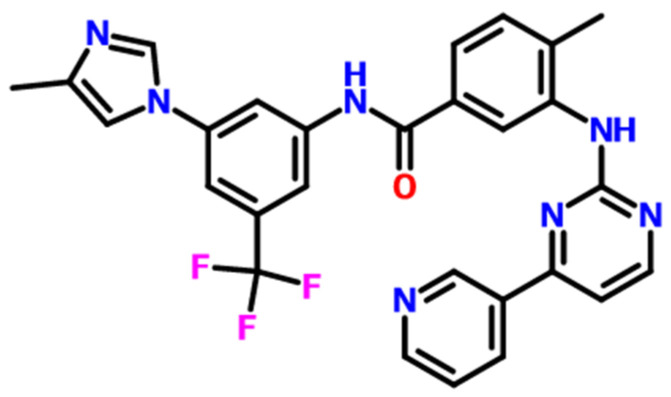
The molecular structure of nilotinib, C_28_H_22_F_3_N_7_O.

Nilotinib was supplied by Anhui Heryi Pharmaceutical Co., Ltd. (Tianchang, China) at 98% purity, characterized as crystalline Form A, with its molecular structure illustrated in Figure 1. Analytical-grade hydrochloric acid (HCl) and sodium hydroxide (NaOH) were both sourced from Shanghai Titan Technology Co., Ltd. (Shanghai, China) at 99% purity. Ultrapure deionized water was prepared using a Millipore water purification system (Applied Membranes Inc., Vista, CA, USA).

### 2.2. Methods

#### 2.2.1. Neutralization Precipitation

**Figure 2:**
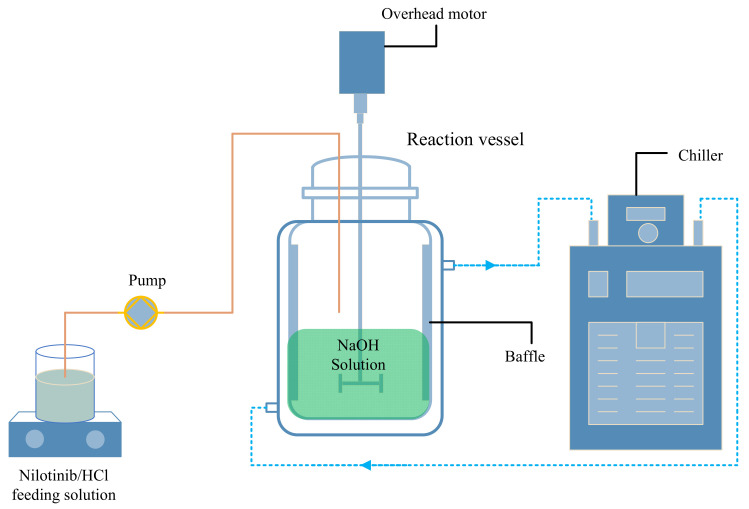
Experimental setup for the preparation of amorphous nilotinib.

Figure 2 illustrates the scale investigation, in which solid nilotinib (5, 25, 50, 75, and 100 g) was fully solubilized in 2.0 mol/L HCl solutions using corresponding solvent volumes of 75, 375, 750, 1125, and 1500 mL. This systematic approach produced nilotinib hydrochloride solutions with a consistent concentration of 0.126 mol/L across all scales. In a separate investigation concerning the concentration of nilotinib, solutions were prepared with concentrations of 0.042, 0.084, 0.126, and 0.168 mol/L. The dissolution process was conducted at a controlled temperature of 25 °C in a water bath (WB100-2F, JOANLAB, Huzhou, China). The filtrate containing nilotinib hydrochloride was first clarified through a 30–50 μm qualitative filter paper (TY3-0005, Shanghai Titan Technology, Shanghai, China) to remove particulate impurities. For neutralization, a stoichiometric NaOH solution matching the initial HCl concentration was prepared and transferred to a four-baffle mixing vessel (S300, Beijing Century Senlang, Beijing, China). The system maintained at 1000 rpm agitation received continuous addition of the nilotinib hydrochloride solution via peristaltic pumping (BT100FC, Baoding Rongbai, Baoding, China) at a 500 mL/min flow rate, facilitating controlled precipitation under precise pH adjustment conditions.

The limited aqueous solubility of nilotinib led to the immediate precipitation of solid particles upon mixing the hydrochloride solution with NaOH, forming a heterogeneous suspension. This system underwent continuous agitation and was allowed to mature for 5 min post-neutralization. Separation of the wet filter cake was achieved through vacuum filtration (SHZ-III A, Gongyi Ruide, Gongyi, China) using Buchner funnels of 6, 15, 18, and 20 cm diameters (Shanghai Titan Technology, Shanghai, China). Filtration dynamics were quantified by measuring the time required for complete filtrate passage, subsequently calculating the filtration rates through suspension volume-to-time ratios. Experimental filtration rates demonstrated size-dependent variation at 14, 76, 124, and 158 mL/min for the respective funnel diameters. Residual NaOH removal was performed by rinsing the cake with deionized water during filtration. Thermal dehydration was followed at 40 °C for 18 h in a vacuum oven (DHG-9055A, Wujiang Yonglian, Wujiang, China) before storing the dried material in sealed glass containers at an ambient temperature (25 °C) for 48 h before characterization.

#### 2.2.2. Characterization

##### Powder X-Ray Diffraction (PXRD)

The PXRD analysis of dried samples was performed at room temperature using a Bruker D8 diffractometer (Bruker, Karlsruhe, Germany) under the following conditions: Mo Kα radiation (λ = 0.71073 Å) at 100 kV and 80 mA, with a step size of 0.02° and scan rate of 6°/min over a 2θ range of 5–40°.

##### Differential Scanning Calorimetry (DSC)

DSC analysis of the dried samples was performed using a Q2000 instrument (TA Instruments, New Castle, DE, USA) over a temperature range of 30–300 °C at a heating rate of 10 °C/min. Samples (~5 mg) were hermetically sealed in T161116 pans (TA Instruments, New Castle, DE, USA) and analyzed under a nitrogen atmosphere at a 50 mL/min purge gas flow. The thermal curves enabled the determination of the glass transition temperature (T_g_), crystallization temperature (T_c_), and melting temperature (T_m_) for each formulation. Subsequently, the reduced crystallization temperature (R_c_) value was computed using the specified formula, as shown below [21]. The R_c_ value serves as an indicator of the requisite temperature above the T_g_ for effective crystallization and evaluates the crystallization propensity of amorphous solids.

$$R_{c}=\frac{T_{c}-T_{g}}{T_{m}-T_{g}}$$

##### Thermogravimetric Analysis (TGA)

Water content analysis of dried samples was performed via TGA using a TGA8000 instrument (PerkinElmer, Waltham, MA, USA). Experiments employed open aluminum pans containing ~5 mg of nilotinib sample, subjected to a dynamic nitrogen atmosphere at a 20 mL/min purge flow. The thermal protocol involved heating from 30 to 150 °C at a constant rate of 10 °C/min, with the mass loss data recorded to determine the moisture content.

##### Focused Beam Reflectance Measurement (FBRM)

Continuous monitoring of the precipitate particle size was conducted using an FBRM G400 probe (Mettler Toledo, Columbus, OH, USA) directly immersed in the reaction vessel. Particle size data were acquired at 10 s intervals throughout the feeding process via real-time measurements.

#### 2.2.3. Pair Distribution Function (PDF)

The PDF analysis was conducted via Fourier transformation of PXRD data using PDFgetX2 software (version 1.0, Columbia University, New York, NY, USA), with detailed configuration parameters outlined in reference [22] and the PDFgetX2 user manual. The data format was set to two-theta, output type to gr, wavelength to 0.71073 Å, composition to C_28_H_22_F_3_N_7_O, q_max-inst_ to 26.5, q_max_ to 26, r_min_ to 0.0, r_max_ to 30, and r_step_ to 0.01. The background file utilized the empty sample stage’s PXRD data, while the input file employed the obtained sample’s PXRD data. The software is accessible at https://web.pa.msu.edu/cmp/billinge-group/programs/PDFgetX2/ (accessed on 18 July 2025).

#### 2.2.4. Principal Component Analysis (PCA)

This study employed PCA to analyze variations in PXRD and PDF data across different solid samples. Before PCA, PDF datasets underwent standard normal variate (SNV) transformation to normalize intensity variations unrelated to compositional differences, followed by mean centering. To minimize interference from short-range structural correlations, PDF data within the 0–15 Å range were excluded. All preprocessing, scaling, and PCA analyses were performed using SIMCA 15 software (Version 15.0, Sartorius, Göttingen, Germany). During PCA, the software automatically calculated the goodness-of-fit parameter (R^2^), which measures data conformity to the model, and the predictive validity metric (Q^2^), assessed via cross-validation and indicative of the model’s generalizability to new data [23].

#### 2.2.5. Accelerated Stability Test

Two amorphous nilotinib samples, S_1_ (0.126 mol/L) and S_2_ (0.042 mol/L), were selected for accelerated stability testing. Following drying, each sample was divided into three equal portions. One portion from each sample was immediately analyzed (designated 0 (S_1_) and 0 (S_2_)), while the remaining portions were stored in open glass bottles at 40 °C and 75% relative humidity for 3 and 6 months, yielding samples 3 (S_1_), 6 (S_1_), 3 (S_2_), and 6 (S_2_). PXRD, PDF, and PCA analyses were performed on all samples post storage following the procedures detailed in Section 2.2.2, Section 2.2.3 and Section 2.2.4. Each experiment was conducted in triplicate using a YP-250GSP stability test chamber (Shanghai Suying Test Instrument Co., Ltd., Shanghai, China).

## 3. Results and Discussion

### 3.1. Optimization of the Concentration of Nilotinib

#### 3.1.1. PDF and PCA of PXRD for the Optimization of the Concentration of Nilotinib

**Figure 3:**
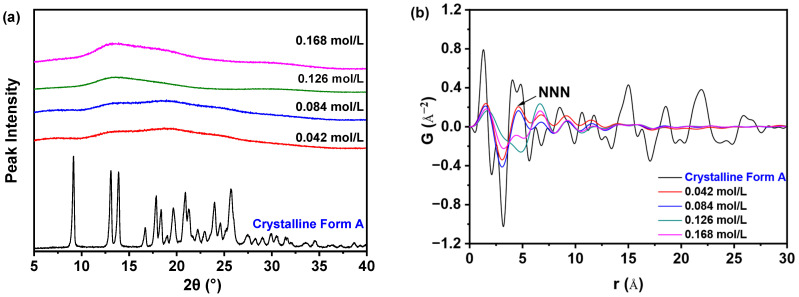
PDF analysis on PXRD data for investigating effects of nilotinib concentration: (**a**) PXRD patterns; (**b**) PDF trace (the mass of nilotinib was 50 g, and the filtration rate was 124 mL/min).

Figure 3a demonstrates that varying the concentration of nilotinib results in samples that all exhibit only a halo diffraction line, which is characteristic of an amorphous state. This observation stands in stark contrast to the crystalline Form A of nilotinib, which serves as a reference. However, the differentiation of various amorphous solid samples through PXRD patterns presents challenges. To enhance the understanding of the distinctions between different amorphous solids, PDF analysis was utilized to interpret the PXRD data, as depicted in Figure 3b. In the PDF trace analysis for nilotinib, a peak at an atomic distance of r = 4.62 Å was identified as the next nearest neighbor (NNN) peak, which corresponds to the crystal lattice height of nilotinib in its crystalline Form A [15,24]. This methodology has also been applied to other drug compounds, such as indomethacin, where the NNN peak corresponds to the molecular coordination sphere [25]. The PDF has proven to be an effective instrument for the structural characterization of amorphous solids [16,17,26]. Prior studies have indicated that the G_NNN_ value serves as a reliable metric for assessing the degree of disorder within amorphous materials [11,12,13,14,15]. Therefore, it is noteworthy that the solid state of nilotinib precipitated at a concentration of 0.126 mol/L exhibited more pronounced variations in the PDF trace and demonstrated the lowest peak height for the NNN peak when compared to amorphous solids generated at other nilotinib concentrations (0.042, 0.084, and 0.168 mol/L).

**Figure 4:**
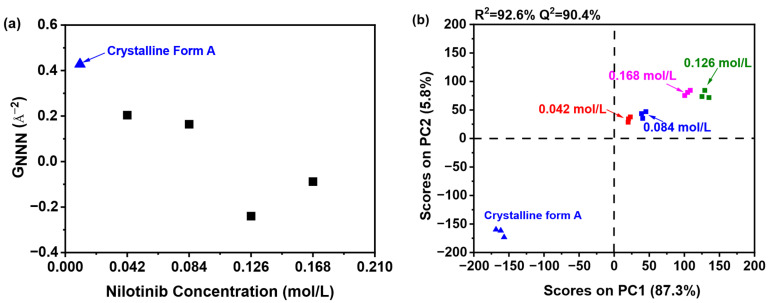
PCA on PDF data for investigating effects of nilotinib concentration: (**a**) G_NNN_ value; (**b**) PCA scores plot (the mass of nilotinib was 50 g and the filtration rate was 124 mL/min).

As illustrated in Figure 4a, the sample precipitated at a nilotinib concentration of 0.126 mol/L exhibited the lowest G_NNN_ value in comparison to those precipitated at concentrations of 0.042, 0.084, and 0.168 mol/L. Additionally, only a slight variation in G_NNN_ values was noted between the samples precipitated at 0.042 and 0.084 mol/L. These findings imply that samples obtained at the optimal nilotinib concentration of 0.126 mol/L demonstrate a greater degree of disorder relative to those produced at concentrations that are either lower or higher. As previously noted, samples exhibiting a higher degree of disorder may demonstrate enhanced physical stability [15]. Consequently, it was determined that the amorphous sample prepared with a nilotinib concentration of 0.126 mol/L possesses the greatest physical stability.

To further elucidate the differences among the samples, PCA was conducted using the previously mentioned PDF data, as illustrated in Figure 4b. The analysis demonstrated that the first principal component (PC1) explained over 87.3% of the total variance, while the second principal component (PC2) contributed 5.8% to the variation. PC1 was identified as the primary factor influencing whether the reference crystalline Form A sample and the precipitated amorphous materials exhibited congruent or divergent PDF patterns during structural characterization. Furthermore, the findings indicated a strong correlation (R^2^ = 92.6%), suggesting significant agreement between the observed and predicted values. The predictive accuracy (Q^2^) was determined to be 90.4%, thereby affirming the high reliability of the model in forecasting future outcomes.

A comprehensive comparative analysis was conducted to evaluate samples prepared with different nilotinib concentrations against the reference crystalline Form A. The investigation revealed four distinguishable categories based on their molecular characteristics. The first and most fundamental category consisted of the crystalline Form A itself, serving as the benchmark for comparison. The second category included two samples prepared with relatively low nilotinib concentrations of 0.042 and 0.084 mol/L, which showed similar molecular patterns. Moving to higher concentrations, the third category comprised samples prepared with 0.168 mol/L of nilotinib, demonstrating distinct properties from both lower and intermediate concentrations. Most remarkably, the fourth category contained only the sample prepared with 0.126 mol/L of nilotinib, which stood out significantly from all other preparations. The PC1 plot provided particularly insightful results, showing a pronounced separation between the 0.126 mol/L sample and those prepared at other concentrations. This substantial divergence suggests that among all the concentration variations tested, the 0.126 mol/L formulation exhibited the most distinct molecular disorder profile relative to the reference crystalline Form A. The degree of separation observed in the PC1 plot indicates that this specific concentration may induce unique molecular arrangements or crystalline packing patterns that differ substantially from both lower and higher nilotinib concentrations. These findings highlight the concentration-dependent nature of nilotinib’s molecular organization and suggest that 0.126 mol/L represents a critical threshold where significant structural modifications occur in the crystalline formation process.

The neutralization precipitation method for amorphous solids is significantly affected by the solute concentration, impacting physical stability. Higher concentrations promote supersaturation and also increase crystallization risk during storage due to elevated chemical potential [27]. Additionally, high concentrations yield more porous particles with a greater surface area, improving initial precipitation but raising hygroscopicity and oxidation risks. Moisture absorption may cause swelling or phase transitions, while increased surface area accelerates oxidation, compromising stability [28]. Thus, optimizing the solute concentration is crucial in balancing formation kinetics with long-term stability. Therefore, in this study, the sample prepared with a nilotinib concentration of 0.126 mol/L was deemed an optimal concentration, effectively mitigating the potential risks associated with excessive dosing while ensuring robust physical stability.

#### 3.1.2. R_c_ Analysis of DSC Data for the Optimization of the Concentration of Nilotinib

**Figure 5:**
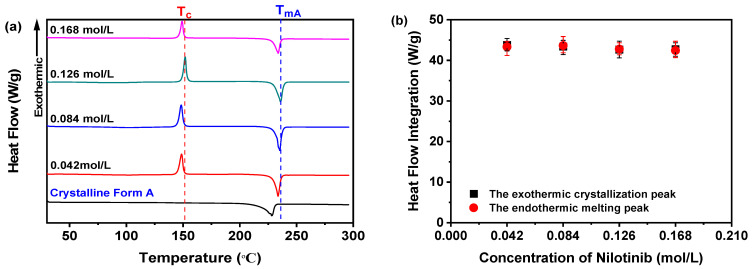
DSC analysis for investigating effects of nilotinib concentration: (**a**) DSC curves; (**b**) heat flow integration for crystallization and melting peaks (the mass of nilotinib was 50 g and the filtration rate was 124 mL/min).

DSC was employed to corroborate the findings derived from the preceding PXRD analysis. As shown in Figure 5a, regardless of concentration, all nilotinib samples consistently displayed a sharp endothermic peak at 235 °C (T_mA_), aligning with the melting point of crystalline Form A, as documented in prior studies [29,30]. Moreover, an exothermic feature near 150 °C was observed, attributed to the crystallization of the amorphous phase into Form A, aligning with previously reported T_c_ values for nilotinib [11,12,13,14]. Moreover, the integration of heat flow between the crystallization and melting peaks exhibited consistency across all four samples analyzed, thereby affirming their entirely amorphous characteristics (Figure 5b). This conclusion was consistent with the PXRD results (Figure 3a), which showed no residual crystallinity in the amorphous samples.

**Figure 6:**
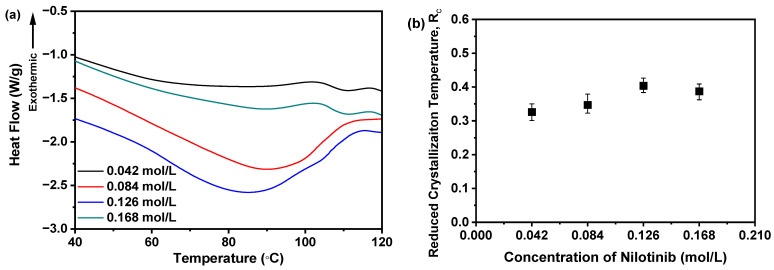
R_c_ analysis on DSC data for investigating effects of nilotinib concentration: (**a**) Glass transition temperature, T_g_; (**b**) reduced crystallization temperature, R_c_ value (*n* = 3) (the mass of nilotinib was 50 g and the filtration rate was 124 mL/min).

Furthermore, it is critical to highlight that the T_g_ of all samples remains undetectable in Figure 5a. To address this, the DSC thermograms within the 40–120 °C range were magnified, as illustrated in Figure 6a. Subsequent examination indicated no discernible correlation between the nilotinib concentration and T_g_ values across the four samples. These results imply that variations in nilotinib concentration do not substantially modulate the T_g_ of the samples studied.

A higher R_c_ value indicates that the sample has a stronger resistance to the transition from amorphous to crystalline form. The findings indicate that the sample with a higher R_c_ value demonstrates improved physical stability [21,31]. Consequently, as depicted in Figure 6b, the R_c_ value is influenced by the concentration of nilotinib. The sample precipitated with 0.126 mol/L of nilotinib demonstrated the highest R_c_ value. In contrast, samples prepared with 0.168 mol/L nilotinib showed a lower R_c_ value than those with 0.126 mol/L, indicating reduced physical stability compared to the 0.126 mol/L samples. Moreover, a reduction in the concentration of nilotinib from 0.084 to 0.042 mol/L also led to a decrease in the R_c_ value, indicating a gradual decline in physical stability associated with lower nilotinib concentrations. The study thus confirmed the PCA results, demonstrating that 0.126 mol/L represents the optimal nilotinib concentration for achieving maximum physical stability in amorphous solids.

#### 3.1.3. Filtration Rate Analysis for the Optimization of the Concentration of Nilotinib

**Figure 7:**
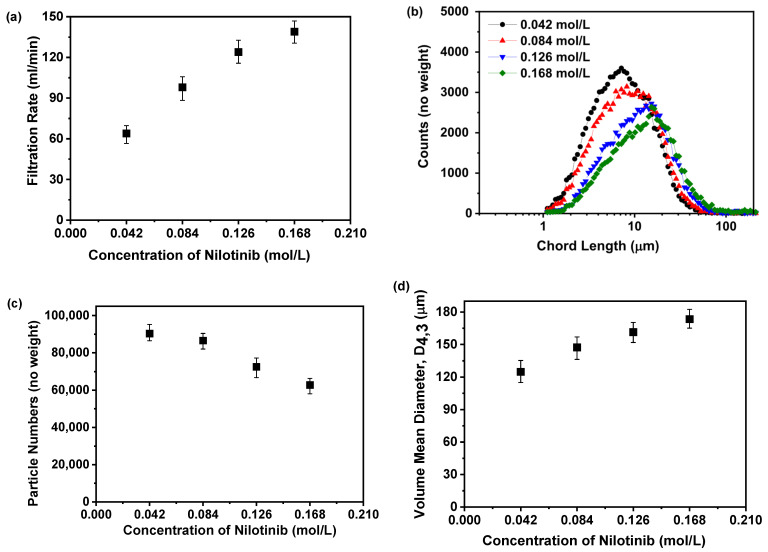
Filtration rate analysis for investigating effects of nilotinib concentration: (**a**) Filtration rate; (**b**) particle size distribution; (**c**) particle numbers; and (**d**) volume mean diameter, D_4,3_ (the mass of nilotinib was 50 g and the filtration rate was 124 mL/min).

Figure 7a reveals a positive correlation between nilotinib concentration and the filtration rate of its precipitated suspension. Specifically, increasing the nilotinib concentration from 0.042 to 0.168 mol/L led to a proportional rise in filtration rates, ascending from 64 to 139 mL/min, thereby demonstrating a direct relationship between the two variables. This observed trend may be attributed to the formation of finer solid particles at lower nilotinib concentrations, which can impede the filtration process, a conclusion that aligns with prior studies [32]. To validate this hypothesis, the particle size distribution was assessed using FBRM, as depicted in Figure 7b,c. The results revealed that lower nilotinib concentrations were associated with smaller particle sizes and a higher particle count. This correlation between nilotinib concentration and particle size was further substantiated by the volume mean diameter (VMD) derived from the FBRM data, as shown in Figure 7d. The analysis indicated a direct relationship between nilotinib concentration and particle size, with an increase from 0.042 to 0.168 mol/L, resulting in a rise in the VMD from 124.85 to 173.39 μm.

Previous studies have shown that particle size and quantity significantly affect separation time [33]. Therefore, precipitation and storage before filtration were limited to 30 min to avoid prolonged processing, which could destabilize amorphous solids by promoting crystallization. Additionally, residual HCl or NaOH in the suspension may induce rapid crystallization if not promptly removed during filtration, as confirmed in prior work [11]. Furthermore, higher nilotinib concentrations increase supersaturation, accelerating nucleation and promoting rapid solute aggregation into more nuclei [12]. Enhanced supersaturation also speeds up solute diffusion and particle growth, forming larger amorphous particles [34]. Additionally, higher solute concentrations strengthen interparticle interactions, increasing aggregation and cluster formation, which further grow into larger amorphous particles [35].

### 3.2. Optimization of the Filtration Rate

#### 3.2.1. PDF and PCA of PXRD for the Optimization of the Filtration Rate

**Figure 8:**
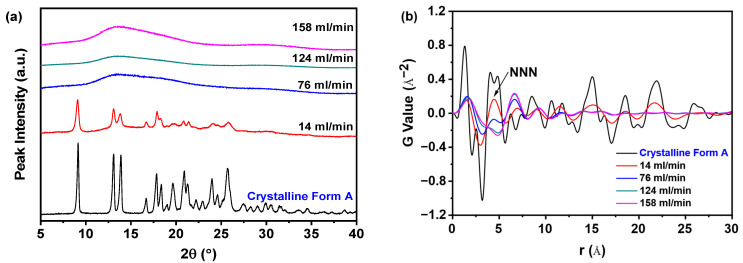
PDF analysis on PXRD data for investigating effects of filtration rate: (**a**) PXRD patterns; (**b**) PDF trace (the mass of nilotinib was 50 g and the concentration of nilotinib was 0.126 mol/L).

Figure 8a indicates that nilotinib obtained at a filtration rate of 14 mL/min formed crystalline Form A, whereas samples filtered between 76 and 158 mL/min consistently displayed amorphous halo patterns. Despite their shared amorphous nature, structural differences emerged through PDF analysis (Figure 8b). The crystalline sample (14 mL/min) exhibited the highest NNN peak intensity and prominent PDF fluctuations, while amorphous samples showed reduced NNN peak heights at elevated filtration rates. This trend suggests that faster filtration promotes greater structural disorder in the amorphous solids, corroborated by diminishing NNN peak intensities with increasing filtration rates.

**Figure 9:**
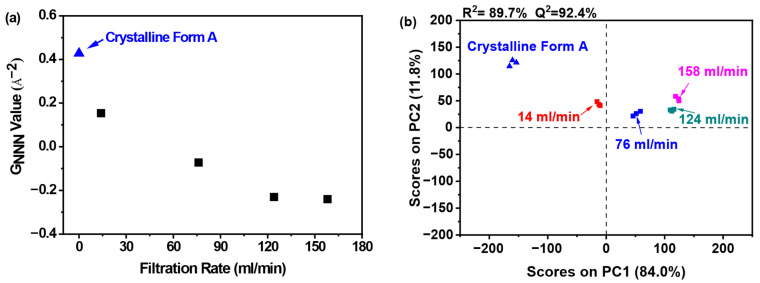
PCA on PDF data for investigating effects of filtration rate: (**a**) G_NNN_ value; (**b**) PCA scores plot (the mass of nilotinib was 50 g and the concentration of nilotinib was 0.126 mol/L).

The structural disorder was further quantified through the G_NNN_ parameter, as depicted in Figure 9a. Notably, samples filtered at 158 mL/min exhibited the minimal G_NNN_ value, which corresponded to the maximal structural disorder observed across all tested filtration rates. This inverse correlation between filtration rate and G_NNN_-derived disorder suggests that elevated processing speeds facilitate increased amorphous domain randomness. The negligible disparity in G_NNN_ values between samples processed at 124 and 158 mL/min indicates that a filtration rate of 124 mL/min is sufficient to induce substantial amorphous disorder. Hence, escalating the filtration rate beyond this threshold is superfluous.

Figure 9b illustrates that PC1 captured over 84.0% of the total variance, with PC2 contributing an additional 11.8%. The robust predictive capability of the model was evidenced by a high cross-validated R^2^ value of 89.7% and Q^2^ value of 92.4%. PCA clustering revealed four distinct sample classifications: (1) crystalline Form A as the reference; (2) samples processed at 14 mL/min; (3) those prepared at 76 mL/min; and (4) samples obtained at elevated filtration rates (124 and 158 mL/min). Notably, the samples prepared at filtration rates of 124 and 158 mL/min displayed significant differences in comparison to the sample prepared at 14 mL/min, indicating structural variations. Moreover, the samples in the fourth category exhibited notable divergence from the reference sample along PC1, suggesting that these samples possessed a robust structure that was distinctly different from that of the reference sample. Furthermore, the samples prepared at a filtration rate of 14 mL/min were similarly positioned at a considerable distance from the reference sample on PC1, reflecting a comparable level of disorder.

Filtration rate fluctuations mechanically impact filter cake formation. Higher rates produce less dense, more porous cakes [36], reducing diffusion resistance for solute transport and enhancing filtration efficiency. Increased porosity may elevate the specific surface area, providing more adsorption sites to stabilize amorphous solids. However, rapid filtration reduces cake compressibility due to insufficient molecular rearrangement time [37]. Therefore, regulating the filtration rate is critical in optimizing the cake structure, compressibility, and physical stability of amorphous solids.

#### 3.2.2. R_c_ Analysis of DSC Data for the Optimization of the Filtration Rate

**Figure 10:**
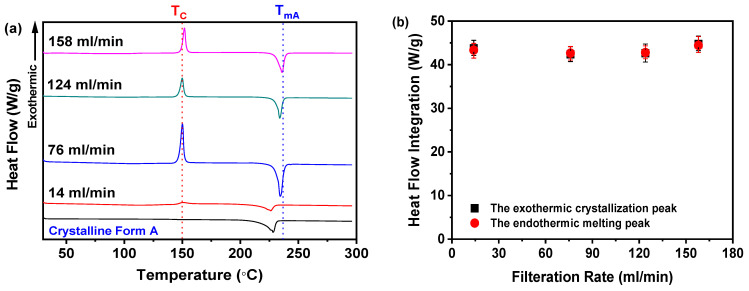
DSC analysis for investigating effects of filtration rate: (**a**) DSC curves; (**b**) heat flow integration for crystallization and melting peaks (the mass of nilotinib was 50 g and the concentration of nilotinib was 0.126 mol/L).

As depicted in Figure 10a, samples obtained at filtration rates between 76 and 158 mL/min exhibited a well-defined endothermic peak at 235 °C, corresponding to the melting point (T_mA_) of nilotinib’s crystalline Form A, consistent with prior studies [29,30]. In contrast, the 14 mL/min sample displayed a DSC thermogram analogous to the crystalline Form A reference. Moreover, all samples, including the 14 mL/min anomaly, presented an exothermic peak near 150 °C, indicative of amorphous-to-crystalline Form A transformation, consistent with reported crystallization temperatures (T_c_) [11,12,13,14]. It is noteworthy that no significant differences in the T_c_ values were observed among the samples collected at different filtration rates, except for the sample prepared at the 14 mL/min filtration rate, which displayed a lower heat flow value compared to those obtained at other filtration rates.

By integrating the heat flows associated with the crystallization and melting events (Figure 10b), the analysis revealed equivalent values for all four samples, confirming their complete amorphous nature. This finding is further substantiated by PXRD data in Figure 8a, which exhibited no characteristic crystalline diffraction peaks.

**Figure 11:**
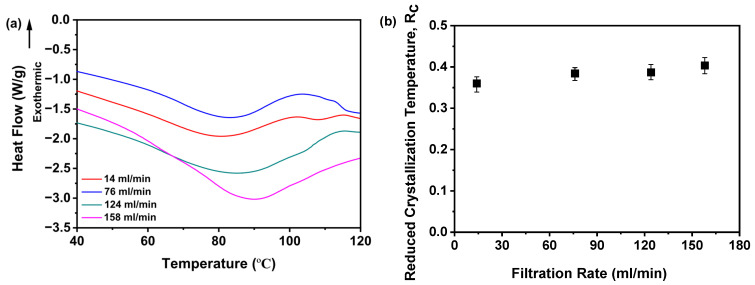
R_c_ analysis on DSC data for investigating effects of filtration rate: (**a**) Glass transition temperature, T_g_; (**b**) reduced crystallization temperature, R_c_ value (*n* = 3) (the mass of nilotinib was 50 g and the concentration of nilotinib was 0.126 mol/L).

Moreover, the T_g_ is systematically characterized in Figure 11a. Notably, samples prepared across a range of filtration rates exhibited a pronounced increasing trend in the T_g_ values correlated with higher filtration rates. As detailed in Table 1, the T_g_ values demonstrated a stepwise increase from 80.72 to 89.93 °C as the filtration rate was elevated from 14 to 158 mL/min. These collective findings robustly demonstrate that the filtration rate exerts a significant influence on the thermal characteristics of the studied materials, as evidenced by the consistent positive correlation between the filtration rate and T_g_.

Furthermore, Figure 11b illustrates the dependence of the R_c_ value on the filtration rate. Notably, samples prepared at 14 mL/min displayed the minimal R_c_ value, whereas those processed at rates surpassing 124 mL/min consistently yielded elevated R_c_ values. Although samples fabricated at 124 and 158 mL/min exhibited marginal discrepancies in R_c_, their near-identical thermal profiles suggest negligible disparities in physical stability. This observation aligns with PXRD evidence, as shown in Figure 8a, reinforcing the amorphous nature of these samples. Mechanistically, the inherently disordered molecular arrangement characteristic of amorphous solids contributes to their elevated energy state. An increase in the filtration rate may exacerbate this disorder, thereby augmenting the degree of molecular disarray and potentially enhancing the physical stability of the amorphous solids. The disordered configuration of molecules poses a barrier to crystal formation, as the latter necessitates specific positional and orientational arrangements of molecules, which a disordered state impedes. Thus, DSC data corroborate that filtration rates exceeding 124 mL/min promote the formation of amorphous solids with enhanced physical stability, as evidenced by their elevated R_c_ values.

#### 3.2.3. TGA for the Optimization of the Filtration Rate

**Figure 12:**
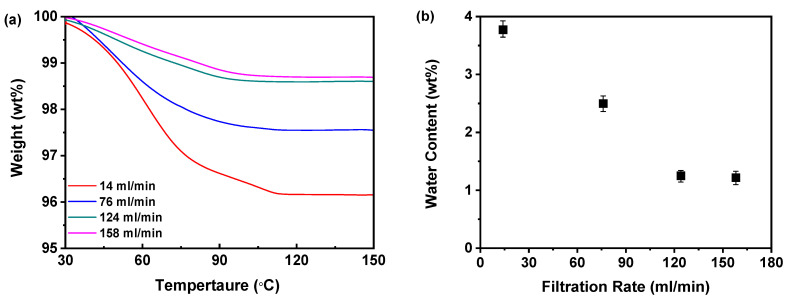
TGA for investigating effects of filtration rate: (**a**) TGA curves; (**b**) water content (*n* = 3) (the mass of nilotinib was 50 g and the concentration of nilotinib was 0.126 mol/L).

Figure 12a demonstrates that the mass of the samples exhibited a gradual decline as the temperature increased to 100 °C during TGA, which can be attributed to the evaporation of moisture present in the samples. However, beyond the 100 °C threshold, the lack of mass loss indicated that the dried samples did not retain any residual moisture. As the filtration rate increased from 14 to 158 mL/min, the mass loss of the sample gradually decreased, suggesting a reduction in residual water content within the samples. Notably, the mass loss observed at filtration rates of 124 and 158 mL/min was nearly equivalent, indicating that a filtration rate of 124 mL/min was adequate to achieve a significant reduction in water content. Further increasing the filtration rate to 158 mL/min did not result in a substantial decrease in the moisture content of the amorphous solids. Additionally, Figure 12b illustrates that the elevation of the filtration rate led to a reduction in water content from 3.78% to 1.22%. While higher filtration rates effectively reduced the moisture content in the dried samples, the difference between the 124 and 158 mL/min rates was minimal, reinforcing the conclusion that a filtration rate of 124 mL/min is sufficient for effective moisture reduction.

Moreover, as shown in Table 1, the T_g_ values of samples obtained at varying filtration rates demonstrated a distinct increasing trend correlated with increased filtration rates. This is highly dependent on the moisture content in the samples. From the above TGA, it can be seen that the slower filtration rate results in a higher moisture content in the samples, and that the increase in moisture content leads to a significant decrease in the T_g_. Notably, the observed differences in water content among the prepared samples cannot be attributed to variations in the drying procedure, as identical drying conditions were rigorously maintained across all samples. Furthermore, given that moisture can act as a plasticizer for amorphous materials, enhancing molecular mobility and promoting crystallization [38], this may explain why a slower filtration rate results in samples with lower physical stability. Although both 124 and 158 mL/min filtration rates are suitable for producing physically stable amorphous solids, the implementation of a 158 mL/min rate necessitates greater investment in scale equipment. Therefore, considering both physical stability and economic factors, a filtration rate of 124 mL/min is recommended as the optimal choice.

### 3.3. Optimization of the Scale of Nilotinib

#### 3.3.1. PDF and PCA of PXRD for the Optimization of the Scale of Nilotinib

The investigation into the scale-up production of drug formulations represents a critical phase in the transition from laboratory methodologies to industrial manufacturing. Scale-up studies facilitate the optimization of process parameters and enable the identification and resolution of engineering challenges that may not be apparent during small-scale experiments, including issues related to equipment compatibility and the stability of raw materials. Furthermore, scale-up production contributes to the reduction in manufacturing costs, enhances the uniformity of product quality, and ensures compliance with regulatory standards in scale production. This process ultimately provides a dependable foundation for the commercialization and clinical application of drugs [39]. Therefore, based on the above optimal neutralization precipitation conditions, including the concentration of nilotinib and the filtration rate, the scale test for the neutralization precipitation of amorphous nilotinib solids was conducted.

**Figure 13:**
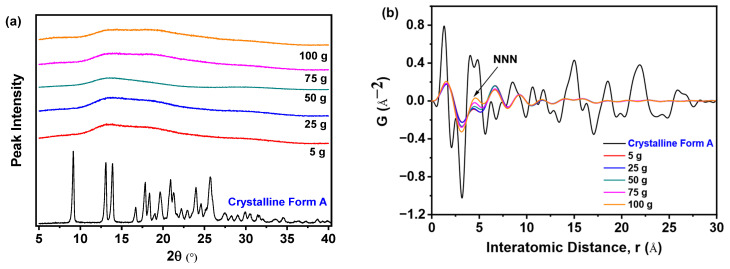
PDF analysis on PXRD data for investigating the effects of scale of nilotinib: (**a**) PXRD patterns; (**b**) PDF traces (the filtration rate was 124 mL/min and the concentration of nilotinib was 0.126 mol/L).

Figure 13a demonstrates that all samples prepared with varying nilotinib quantities uniformly exhibited broad hai lo diffraction patterns in PXRD, confirming the retention of their amorphous characteristics irrespective of the processing scale. This consistent absence of crystalline diffraction peaks across all samples unequivocally validates the maintenance of an amorphous solid state during scale-up. Figure 13b reveals that samples prepared with 75 and 100 g of nilotinib exhibited greater variability in the PDF trace and attained the highest intensity for the NNN peak. In contrast, samples prepared using 5, 25, or 50 g of nilotinib displayed minimal variation in NNN peak height, indicating reduced structural heterogeneity at smaller and intermediate scales.

**Figure 14:**
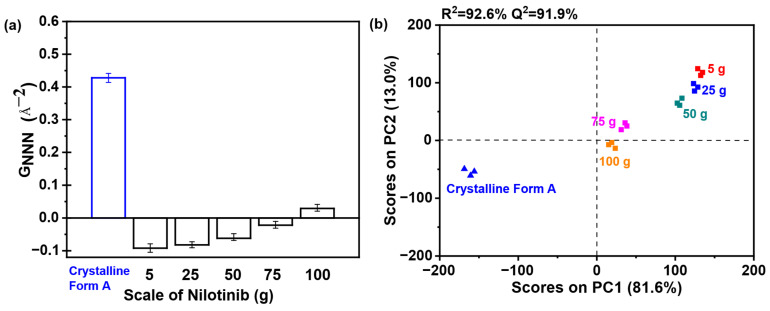
PCA on PDF data for investigating the effects of scale of nilotinib: (**a**) G_NNN_ values (*n* = 3); (**b**) PCA scores plot (the filtration rate was 124 mL/min and the concentration of nilotinib was 0.126 mol/L).

As shown in Figure 14a, the analysis revealed that the samples obtained with 5 to 50 g of nilotinib exhibited lower G_NNN_ values relative to the samples prepared with 75 and 100 g of nilotinib, reflecting heightened amorphous disorder. Consequently, it can be concluded that the samples prepared with 75 and 100 g exhibited a greater propensity for crystallization compared to those prepared with 50 g or less. As depicted in Figure 14b, PC1 accounted for more than 81.6% of the total variance, while PC2 contributed 13.0% to the overall variation. Furthermore, the findings indicated a strong correlation (R^2^ = 92.6%) and a significant level of predictive accuracy (Q^2^ = 91.9%). In addition, the samples were categorized into three distinct groups: (1) crystalline Form A (reference sample); (2) the sample obtained at a scale of 75 and 100 g; and (3) the remaining samples produced at scales of 5 g, 25 g, and 50 g. It is noteworthy that the 100 g samples aligned closely with the PC1 axis, exhibiting pronounced proximity to the crystalline Form A reference, whereas within the amorphous cluster, the 5 g preparation exhibited the greatest deviation from Form A, indicative of a distinct solid-state architecture characterized by heightened configurational disorder.

When preparing amorphous solids via neutralization precipitation, the drug scale critically impacts physical stability. Larger scales elevate local concentrations, increasing crystallization risk by promoting molecular aggregation and reorganization into stable crystalline structures. Scale also affects the amorphous solid’s aggregated structure, thermal stability, and hygroscopicity. Excessive scaling exacerbates hygroscopicity, undermining stability. For nilotinib, results show 50 g scaling maintains stability, while 75–100 g quantities cause significant declines. Thus, a mass of 50 g is determined to be the optimal scale for maintaining physical stability.

#### 3.3.2. R_c_ Analysis of DSC Data for the Optimization of the Scale of Nilotinib

**Figure 15:**
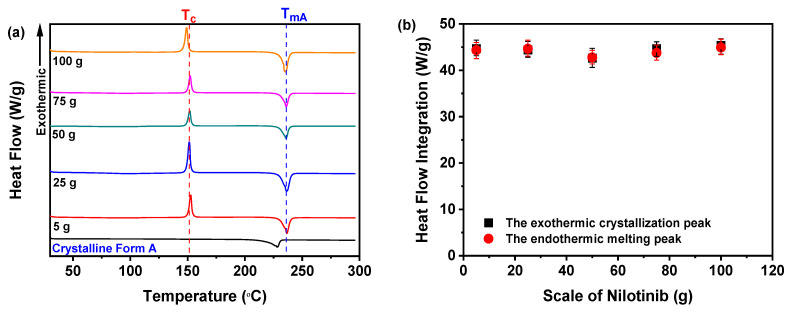
DSC analysis for investigating the effects of scale of nilotinib: (**a**) DSC curves; (**b**) heat flow integration for crystallization and melting peaks (The filtration rate was 124 mL/min, and the concentration of nilotinib was 0.126 mol/L).

As shown in Figure 15a, all nilotinib samples prepared across varying scales exhibited a distinct endothermic peak at 235 °C, corresponding to the T_mA_ of crystalline Form A, consistent with prior reports [29,30]. Additionally, an exothermic peak near 150 °C was observed, aligning with the established T_c_ for the amorphous-to-crystalline Form A transition [11,12,13,14]. A critical analysis of the integrated heat flow during crystallization and melting (Figure 15b) revealed congruent enthalpy values across all five samples, confirming their complete amorphous nature. This finding was corroborated via PXRD analysis (Figure 13a), which lacked crystalline peaks, further validating the absence of an ordered structure in these samples.

**Figure 16:**
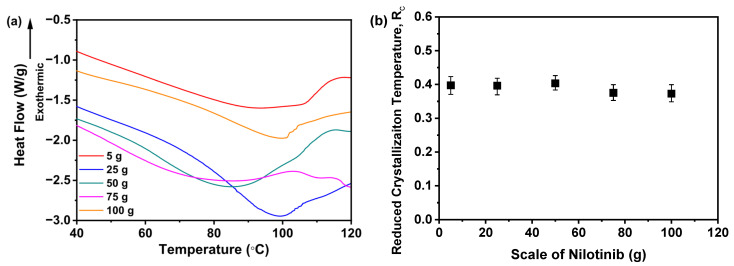
R_c_ analysis on DSC data for investigating the effects of scale of nilotinib: (**a**) Glass transition temperature, T_g_; (**b**) reduced crystallization temperature, R_c_ value (*n* = 3) (the filtration rate was 124 mL/min and the concentration of nilotinib was 0.126 mol/L).

Moreover, Figure 16a illustrates the T_g_ of the samples. Notably, no clear trend was observed in the T_g_ values across varying production scales of nilotinib. Statistical analysis confirmed that differences in scale of nilotinib did not produce a statistically significant effect on the T_g_ of the five tested samples, as further supported by complementary PXRD data. Furthermore, Figure 16b highlights the dependence of the R_c_ value on the processing scale of nilotinib. Remarkably, the 50 g formulation achieved the maximum R_c_ value, signifying optimal physical stability. By contrast, samples fabricated with 5 and 25 g of nilotinib exhibited negligible R_c_ deviations, implying consistent stability properties. Conversely, scaling up nilotinib to 75–100 g triggered a progressive R_c_ reduction, evidencing compromised stability at elevated mass loads. Collectively, these results underscore 50 g as the superior scale for enhancing amorphous solid-state stability.

#### 3.3.3. Filtration Rate Analysis for the Optimization of the Scale of Nilotinib

**Figure 17:**
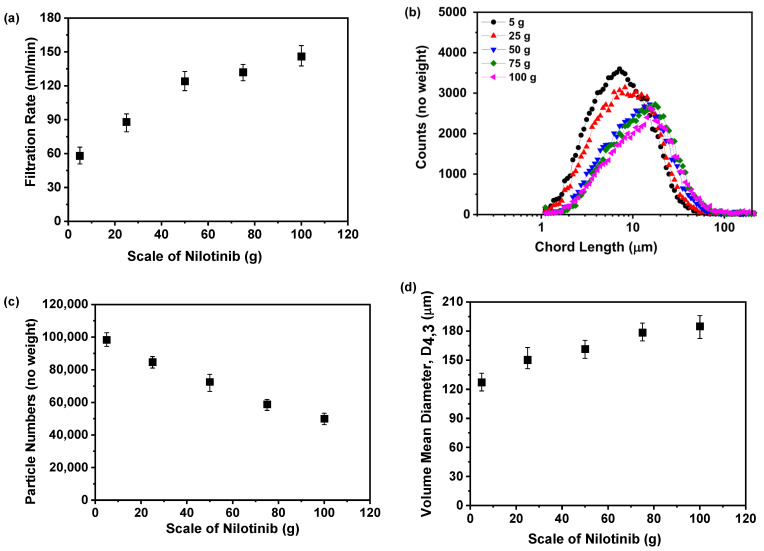
Filtration rate analysis for investigating the effects of scale of nilotinib: (**a**) Filtration rate; (**b**) particle size distribution; (**c**) particle numbers; and (**d**) volume mean diameter, D_4,3_ (the filtration rate was 124 mL/min and the concentration of nilotinib was 0.126 mol/L).

Figure 17a illustrates the direct proportionality between the scale of nilotinib and the filtration rate of the resultant suspension. A progressive enhancement in filtration rate is evident as the drug quantity escalates from 5 to 100 g, with values rising systematically from 58 to 146 mL/min. This dose–response relationship likely arises from particle size dynamics, where reduced-scale operations produce a finer particulate morphology that inherently resists filtration processes, a phenomenon consistent with previous findings on particle-dependent filtration efficiency [32]. To rigorously test this hypothesis, FBRM was employed to characterize the particle size distributions, with experimental configurations illustrated in Figure 17b,c. The results revealed that smaller nilotinib scales were associated with smaller particle sizes and a higher particle count. The dose-responsive particle growth behavior was further corroborated via VMD metrics derived from FBRM analysis, as shown in Figure 17d. Quantitative assessment revealed a robust positive gradient between the scale of nilotinib and particulate dimensions, where dose escalation from 5 to 100 g corresponded to a VMD enhancement from 127.21 to 184.84 μm.

### 3.4. Accelerated Stability Test Results

**Figure 18:**
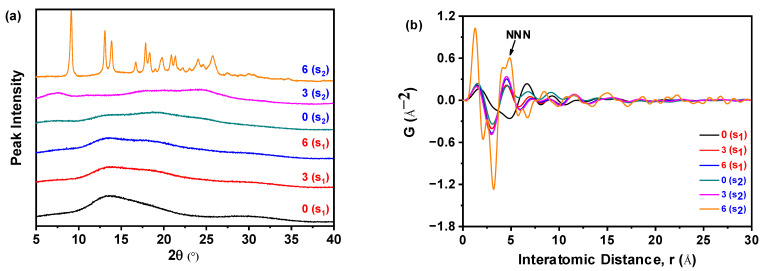
PDF analysis of PXRD data for the accelerated stability test: (**a**) PXRD patterns; (**b**) PDF traces. (0 (S1), 3 (S1), and 6 (S1) refer to the sample S1 that has been preserved for 0, 3, and 6 months, respectively; 0 (S2), 3 (S2), and 6 (S2) refer to the sample S2 that has been preserved for 0, 3, and 6 months, respectively.).

As shown in Figure 18a, PXRD analysis revealed that sample S_1_, obtained at a nilotinib concentration of 0.126 mol/L and stored for 0, 3, and 6 months, consistently exhibited only a halo diffraction pattern. This indicates that S_1_ maintained its amorphous structure without crystallization during the accelerated stability study. In contrast, sample S_2_, prepared at a lower nilotinib concentration of 0.042 mol/L, retained an amorphous halo at 0 and 3 months. However, after 6 months of storage, the PXRD pattern of S_2_ displayed a sharp peak corresponding to crystalline Form A of nilotinib [29,30], indicative of partial crystallization. Quantitative analysis based on prior calibration methods [15] showed that S_2_ comprised 65% amorphous content and 35% crystalline Form A after 6 months, confirming a time-dependent phase transition from amorphous to crystalline Form A between 3 and 6 months. These findings demonstrate that S_1_ exhibited greater physical stability than S_2_, consistent with the results from PDF and R_c_ analyses discussed in Section 3.1. This consistency reinforces the reliability of the analytical approach employed in the study.

The data presented in Figure 18b indicate that sample S_2_, obtained at a nilotinib concentration of 0.042 mol/L and subjected to 6 months of storage, exhibited the most pronounced alterations in PDF analysis, alongside the highest recorded NNN peak intensity among all tested samples. Notably, the amorphous fraction of S_1_ demonstrated a progressive rise in the NNN peak height, correlating with extended storage durations from 0 to 6 months. Similarly, the amorphous component of S_2_ displayed an ascending trajectory in the NNN peak intensity during the initial 3 months of storage. These observations suggest concentration-dependent differences in physical stability, with S_2_ exhibiting greater structural reorganization over time than S_1_.

**Figure 19:**
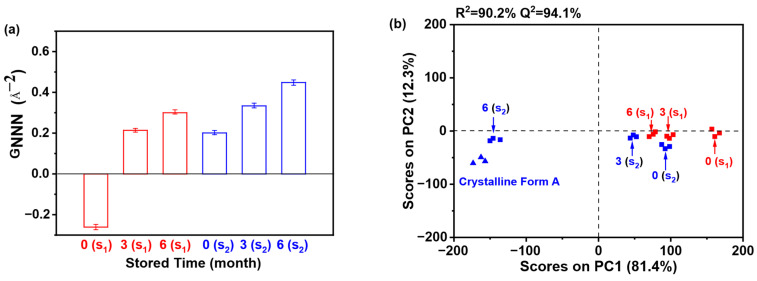
PCA of PDF data for the accelerated stability test: (**a**) G_NNN_ values (*n* = 3); (**b**) PCA scores plot. (0 (S_1_), 3 (S_1_), and 6 (S_1_) refer to the sample S_1_ that has been preserved for 0, 3, and 6 months, respectively; 0 (S_2_), 3 (S_2_), and 6 (S_2_) refer to the sample S_2_ that has been preserved for 0, 3, and 6 months, respectively.).

Moreover, Figure 19a demonstrates that the G_NNN_ values of samples stored for 6 months surpassed those stored for 0 or 3 months, indicating reduced disorder with extended storage. This correlation implies that lower G_NNN_ values correspond to greater structural disorder. Notably, the 0 months sample exhibited the minimal G_NNN_ value, reflecting substantial disorder. When comparing S_1_ and S_2_ (both 0 months of storage), S_1_ showed a lower G_NNN_ value, suggesting heightened disorder and, consequently, enhanced physical stability relative to S_2_. Thus, S_2_ demonstrated a greater propensity for crystallization compared to S_1_, aligning with the findings in Figure 18a. Furthermore, as illustrated in Figure 19b, PC1 and PC2 accounted for 81.4% and 12.3% of the total variance, respectively. The robustness of the model is underscored by high R^2^ (90.2%) and Q^2^ (94.1%) values. PCA enabled the clear classification of samples into four distinct clusters: (1) crystalline Form A (reference); (2) sample S_2_ (6 months storage); (3) sample S_1_ (0 months storage); and (4) other samples. Notably, sample S_2_ (6 months storage) exhibited a close alignment with crystalline Form A along PC1, suggesting a structural similarity to the reference. In contrast, sample S_1_ (0 months of storage) displayed the most significant deviation from crystalline Form A, indicating a unique solid-state architecture and increased disorder. Furthermore, sample S_1_ (0 months of storage) demonstrated a greater deviation from crystalline Form A compared to another 0 months sample (S_2_), thereby reinforcing its distinct stability profile. Collectively, these PCA results affirm that sample S_1_ exhibited superior physical stability relative to sample S_2_.

This research indicates that a 6-month waiting period is not required for the assessment of the physical stability of amorphous pharmaceuticals using PXRD. By utilizing early-stage PXRD analyses alongside PDF and PCA, researchers can efficiently and accurately evaluate disorder and material stability. This comprehensive methodology—integrating the structural insights provided by PXRD, the sensitivity to amorphous domains offered by PDF, and the pattern-recognition capabilities of PCA—presents a high-throughput, precision-oriented alternative to standard accelerated stability tests. As a result, this approach facilitates expedited decision-making and enhances the mechanistic understanding of amorphous drug behavior, thereby exceeding the efficacy of traditional methods that depend on extended storage periods.

### 3.5. Discussion on Future Work

This study focused on investigating the preparation of physically stable amorphous nilotinib without using excipients (e.g., polymers) that could stabilize the amorphous form or enhance solubility. However, it remains unclear whether the obtained amorphous form improves solubility. Additionally, the current preparation scale of nilotinib is limited to laboratory-level production (50 g), which falls far short of industrial-scale manufacturing. Future research should therefore evaluate the solubility enhancement of this physically stable amorphous form compared to crystalline form A of nilotinib, while simultaneously working to scale up the production of amorphous nilotinib.

## 4. Conclusions

A major hurdle in the scale manufacturing of amorphous drugs lies in their thermodynamic instability, which predisposes them to undesirable crystallization and associated declines in solubility. This investigation systematically optimized nilotinib’s neutralization precipitation process by fine-tuning three critical parameters: drug concentration (0.126 mol/L), filtration rate (124 mL/min), and processing scale (50 g). Remarkably, this parameter triad yielded amorphous formulations exhibiting superior physical stability. Methodologically, the study pioneered an integrated analytical platform combining PDF analysis, PCA of PDF data, and R_c_ values from DSC. This multimodal approach accelerated stability assessments while delivering higher precision than conventional accelerated stability protocols. The findings underscore that meticulous parameter control during precipitation directly correlates with improved amorphous stability. More broadly, the validated analytical framework—leveraging PDF/PCA/R_c_ methodologies—establishes a new paradigm for rapid, quantitative stability evaluation. These innovations hold transformative potential for amorphous drug development, enabling the scalable production of physically robust dosage forms. By addressing preparation techniques, characterization methods, stability predictors, and scale-up challenges, this work provides both theoretical principles and practical guidelines applicable across diverse amorphous drug systems.

## Figures and Tables

**Table 1:** The glass transition temperature (T_g_) for samples obtained at different filtration rates.

Numbers	Filtration Rate	Glass Transition Temperature (T_g_), °C	Water Content, %
1	14	80.72	3.78
2	76	83.49	2.50
3	124	86.28	1.25
4	158	89.93	1.22

## Data Availability

Data is contained within the article.
